# Bioprocess development for L-asparaginase production by *Streptomyces rochei*, purification and *in-vitro* efficacy against various human carcinoma cell lines

**DOI:** 10.1038/s41598-020-64052-x

**Published:** 2020-05-14

**Authors:** Noura El-Ahmady El-Naggar, Nancy M. El-Shweihy

**Affiliations:** Department of Bioprocess Development, Genetic Engineering and Biotechnology Research Institute, City of Scientific Research and Technological Applications, Alexandria, Egypt

**Keywords:** Hydrolases, Industrial microbiology

## Abstract

In the near future, the demand for L-asparaginase is expected to rise several times due to an increase in its clinical and industrial applications in various industrial sectors, such as food processing. *Streptomyces* sp. strain NEAE-K is potent L-asparaginase producer, isolated and identified as new subsp. *Streptomyces rochei* subsp. *chromatogenes* NEAE-K and the sequence data has been deposited under accession number KJ200343 at the GenBank database. Sixteen different independent factors were examined for their effects on L-asparaginase production by *Streptomyces rochei* subsp. *chromatogenes* NEAE-K under solid state fermentation conditions using Plackett–Burman design. pH, dextrose and yeast extract were the most significant factors affecting L-asparaginase production. Thus, using central composite design, the optimum levels of these variables were determined. L-asparaginase purification was carried out by ammonium sulfate followed by DEAE-Sepharose CL-6B ion exchange column with a final purification fold of 16.18. The monomeric molecular weight of the purified L-asparaginase was 64 kD as determined by SDS-PAGE method. The *in vitro* effects of L-asparaginase were evaluated on five human tumor cell lines and found to have a strong anti-proliferative effects. The results showed that the strongest cytotoxic effect of L-asparaginase was exerted on the HeLa and HepG-2 cell lines (IC_50_ = 2.16 ± 0.2 and 2.54 ± 0.3 U/mL; respectively). In addition, the selectivity index of L-asparaginase against HeLa and HepG-2 cell lines was 3.94 and 3.35; respectively.

## Introduction

L-asparaginase is one of the amidase group, that catalyzes the hydrolysis of L-asparagine and releases L-aspartic acid and ammonia^1^. L-asparaginase is an effective therapeutic enzyme used in combination with other drugs for treating melanosarcoma, reticulosarbom, lymphocytic leukemia, Hodgkin disease, chronic lymphosarcoma, acute myelomonocytic leukemia, acute myelocytic leukemia and acute lymphoblastic leukemia in children and adults^2,3^. In pediatric oncology, L-asparaginase is the recommended drug used for acute lymphoblastic leukemia therapy, resulting in a total recovery of more than 90 percent of kids within a period of four weeks^3^. The anti-leukemic impact of L-asparaginase based on the fact that the tumor cells need a huge quantity of L-asparagine to survive. The tumor cells cannot synthesize L-asparagine for their needs because of the lack of L-asparagine synthetase and thus cannot synthesize the necessary proteins that rely on L-asparagine^4^. The survival and growth of tumor cells rely on the exogenous source of L-asparagine (consumed in a diet, absorbed and accessible in the circulating pool)^5^. Thus, injection of the enzyme into cancer patients intravenously decreases L-asparagine blood levels and selectively damages tumor cells^6^. Conversely, the normal cells are able to synthesize L-asparagine, thus protecting themselves from L-asparagine starvation^7,8^. Therapeutic long-term use of L-asparaginase induces hypersensitivity reactions^9^. L-asparaginase therapy is associated with a vast array of adverse side effects, like neurological seizures, diabetes, pancreatitis, leucopoenia, hepatotoxicity, fever, skin rashes and abnormal coagulation tests that could result in haemorrhage or intracranial thrombosis^8^.

L-asparaginase is used in the food industry to catalyze the hydrolysis of L-asparagine, and forms L-aspartate as well as ammonia. Therefore, L-asparaginase can be used before frying or baking foods containing both L-asparagine and carbohydrates cooked over 120 °C to reduce the formation of acrylamide^10^ that has been reported to be a major toxic agent and cause neurotoxicity in humans. Also, L-asparaginase showed a good anti-oxidant property^11^. In the near future, L-asparaginase production must be increased to several folds due to its potential applications in the food industry in addition to its clinical applications^12^.

The microbial sources of L-asparaginase have proven to be highly efficient and inexpensive. The ease of cultivation of microorganisms facilitated large-scale production of the enzyme^13^. The most commonly used technique for production of L-asparaginase was stated to be the submerged fermentation (SF). However, it has been shown to have several disadvantages such as elevated costs with low product concentration, excessive effluent generation, excessive treatments and disposal of wastewater in large quantities^14^. In recent years, SSF (solid state fermentation) technique is a promising alternative to submerged fermentation for the production of enzymes, as the production of enzymes is much more than that of submerged fermentation^15^. SSF process also has many additional advantages such as more concentrated enzyme preparations, ease of cultivation, reduced risk of bacterial contamination, easier extraction of the product^16^, low energy demands, decreased generation of wastewater and decreased environmental issues concerning waste disposal^17^.

The composition of fermentation medium and culture conditions (i.e., nutrients, temperature, pH, etc.) play an incredible role in L-asparaginase production^18^. There is no specific medium for maximum L-asparaginase production by different microorganisms. Since every organism has its own requirements to achieve the maximum production of L-asparaginase, the development of suitable medium ingredients and environmental conditions is mandatory. Various environmental conditions and medium ingredients required for microbial growth and enzyme production should be optimized as the conditions of culture which promote the production of enzyme varies greatly with the microorganism’s nature^19^. The conventional strategy for media optimization (one variable-by-time optimization) not only takes a long time, but also ignores the effects of variables interactions and leads to misinterpretation of the results^20^. The statistical designs of experiments were used over many decades and can be achieved in two major steps: firstly, screening of the significant variables and secondly, optimization of those variables^21^. These designs have several advantages, including lesser experiments, which are appropriate for multiple variable experiments; quantify possible interactions between different variables, and finding of the best possible conditions for the maximum response^22^.

Currently, the therapeutic formulations of L-asparaginase, which are widely used for the leukemia therapy, are derived from *E. coli* and *Erwinia chrysanthemi*^23^. It is well known that different preparations for L-asparaginase exhibit different pharmacological and biochemical characteristics and also different side-effects on the normal human cells. This is due to different structural, physicochemical and kinetic properties of L-asparaginase obtained from various biological sources, including microbial sources^24^. Therefore, it is important to explore new sources for L-asparaginase production. Actinomycetes are known to be a less explored source of L-asparaginase^25^. In this study, *Streptomyces rochei* subsp. *chromatogenes* NEAE-K isolated from a soil sample was used for production of glutaminase free L-asparaginase. A statistical approach was used to define the significant variables affecting the production of L-asparaginase and to optimize these variables. Purification and assessment of its antitumor activity against various human carcinoma cell lines were also performed.

## Results and Discussion

In this study, the medium ingredients and environmental conditions that play a critical role in maximizing the production of L-asparaginase have been optimized. The critical factor for solid state fermentation (SSF) is the choice of the appropriate solid substrate for fermentation. Process economization of solid state fermentation aiming at increasing L-asparaginase production by using cheaper materials to reduce the production costs. The soybean substrate was the perfect for L-asparaginase production and this could be explained by the fact that there are adequate quantities of carbohydrates, proteins, lipids and minerals^26^. El-Naggar *et al*.^26^ have reported that soybean was used as substrate for production of L-asparaginase under SSF conditions by *Streptomyces brollosae* NEAE-115.

**Figure 1:**
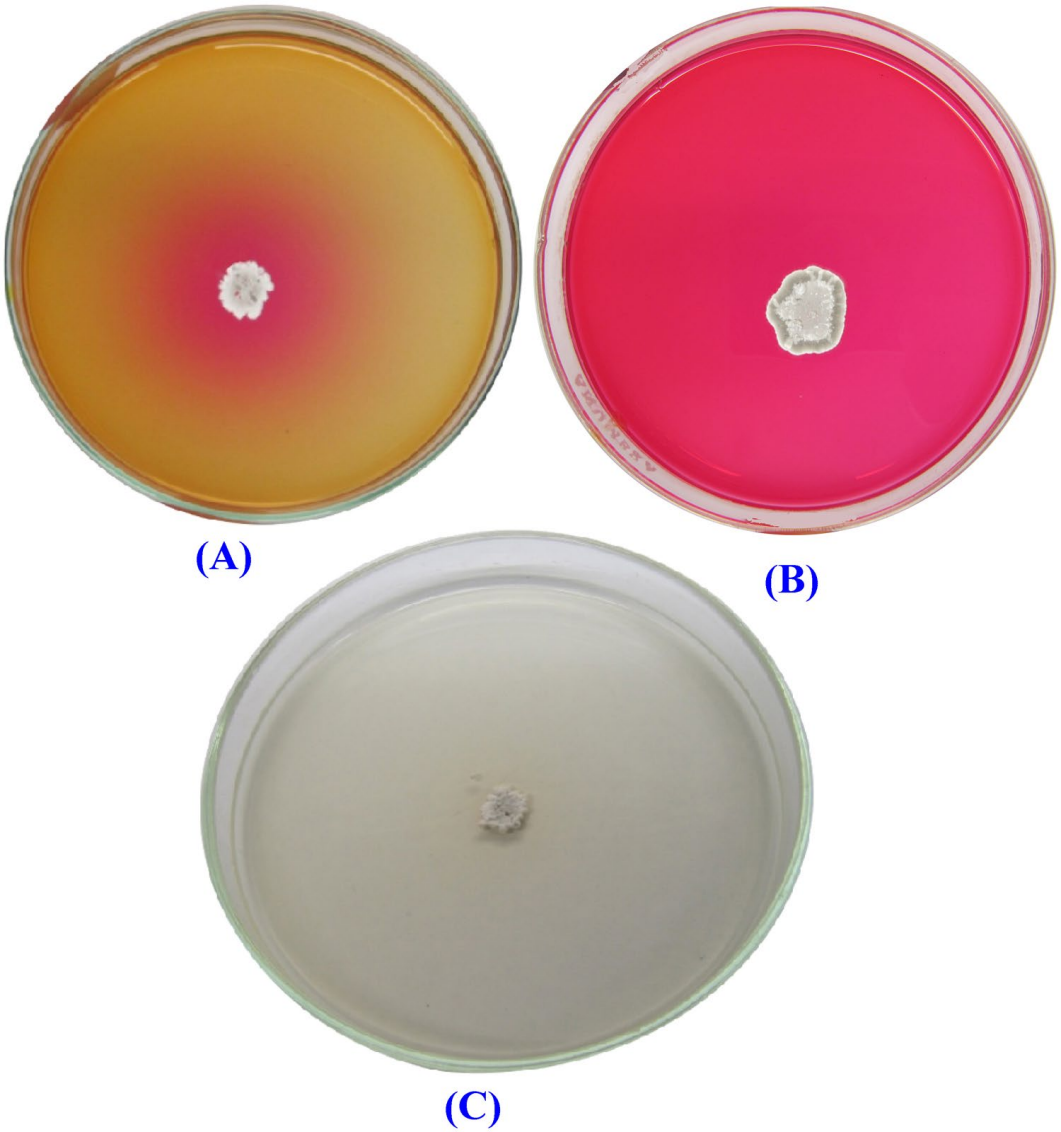
L-asparaginase activity of *Streptomyces* sp. strain NEAE-K detected by plate assay (**A, B**) production of the enzyme indicated by color change in the medium from yellow to pink zone surrounding the colony after two and five days; respectively (**C)** Control plates were prepared as medium without dye.

**Figure 2:**
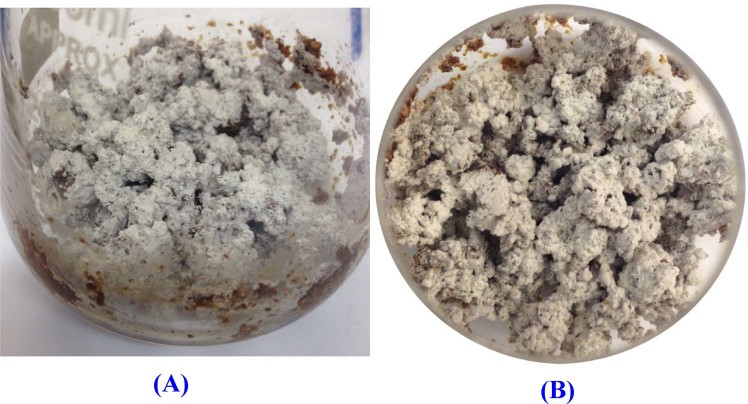
*Streptomyces* sp. strain NEAE-K growth under solid state fermentation conditions after inoculation and incubation for 7 days at 30 °C. (**A**) side view of the flask, (**B**) upper view.

Several strains of actinomycetes were isolated from soil samples collected from Egypt and Saudi Arabia and evaluated for their L-asparaginase activities using the plate assay method. The presence of L-asparaginase activity was detected by the formation of pink areas around the colonies (Fig. 1). Among the tested strains, *Streptomyces* sp. strain NEAE-K is promising and was therefore selected for further studies. L-glutaminase activity was determined for the selected strain and the results demonstrated that the produced enzyme is glutaminase free L-asparaginase. In our previous study^26^, a comparative study was performed to assess the suitability of soybean and wheat bran for L-asparaginase production as carbon sources whether individually or mixed. It was clear from the results that the two substrates support L-asparaginase production (wheat bran; 16.49 U/gds and soybean meal; 21.84 U/gds). However, maximum L-asparaginase production under SSF was achieved with mixed soybean and wheat bran (1:1; w/w) (44.821 U/gds) (Fig. 2). Isaac and Abu-Tahon^27^ reported that of the seven natural substrates, wheat bran was the most potential substrate used by *Fusarium solani* AUMC 8615 to produce maximum L-asparaginase activity of 187.9 U/mL.

### Morphological and cultural features of the strain NEAE-K

**Figure 3:**
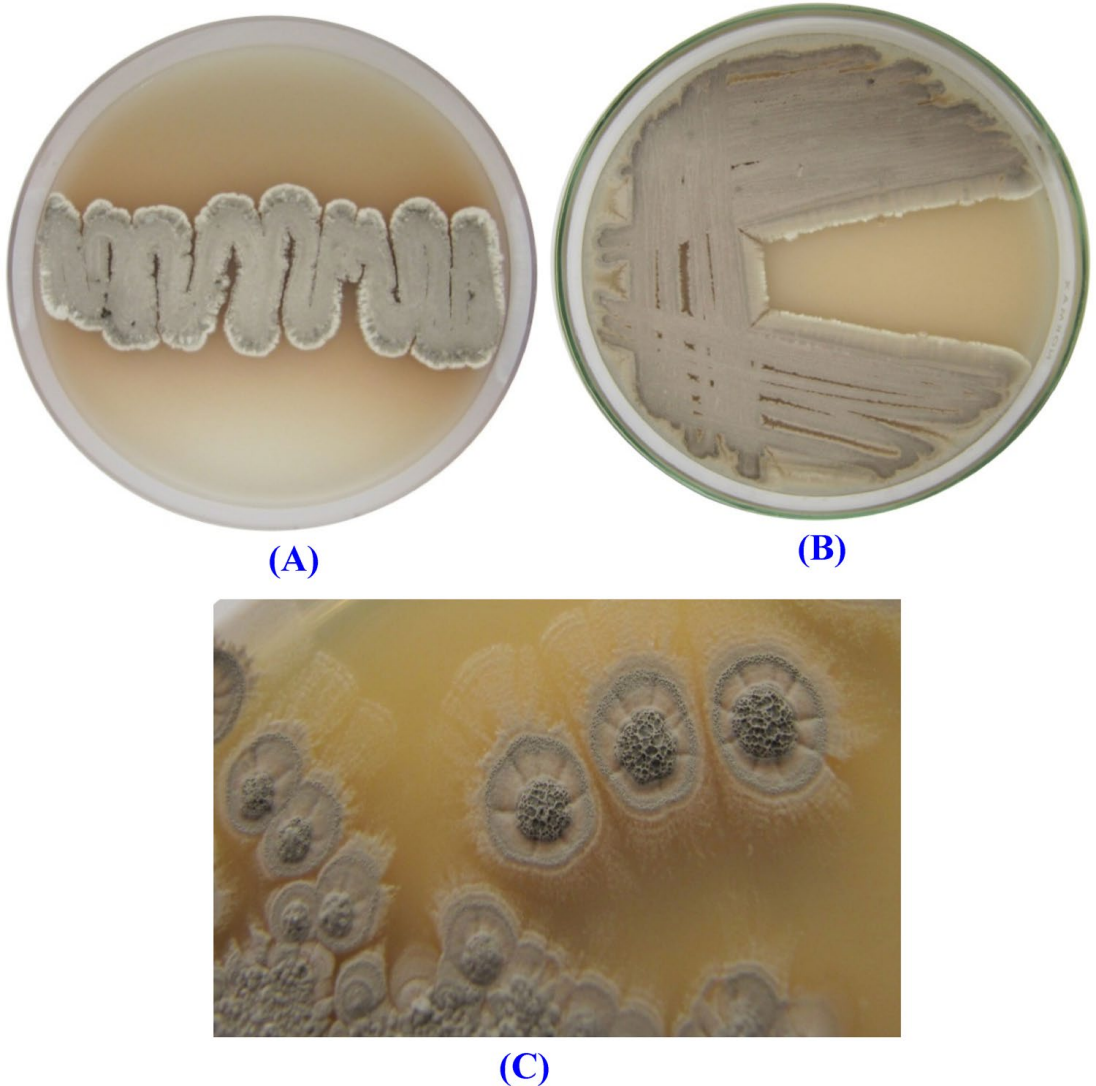
Color of the aerial mycelium of *Streptomyces* sp. strain NEAE-K grown on: (**A,B**) inorganic salt-starch agar and (**C**) yeast extract -malt extract agar for 7 days of incubation at 30 °C.

**Figure 4:**
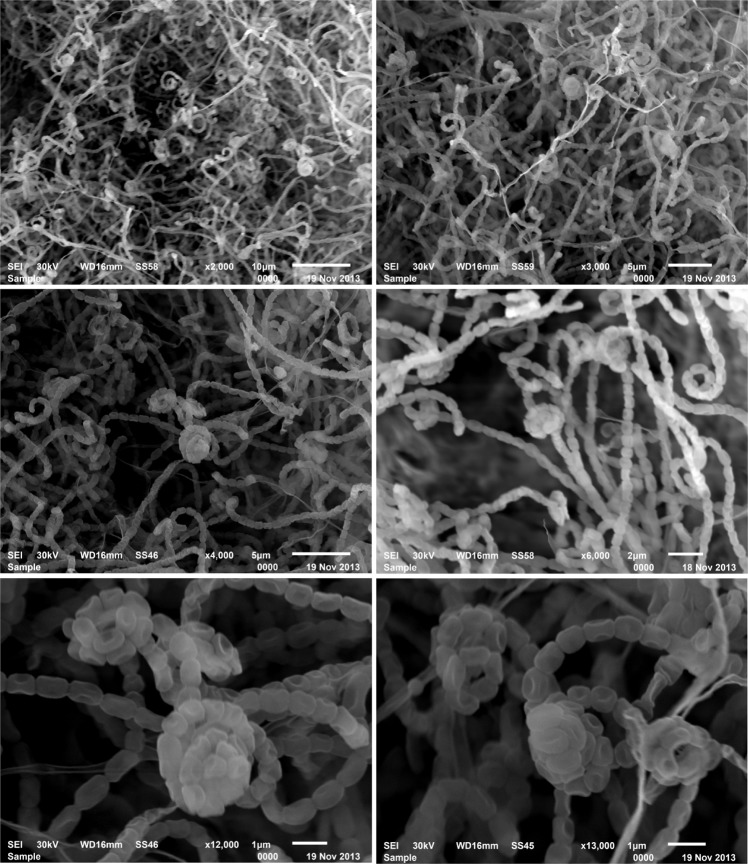
Scanning electron micrograph showing the spore-chain morphology of strain NEAE -K at magnification of 2000X–13000X.

The morphological features of the strain NEAE-K are compatible with the genus *Streptomyces*^28^. Strain NEAE-K grew well on all tested ISP media 2-7. The organism is aerobic and mesophilic. Color of the aerial mycelium was gray on all media tested (Fig. 3, Table 1). Colony reverse side has brown pigments on oatmeal agar, yeast-malt agar, inorganic salts-starch agar and glycerol-asparagine agar media. Faint brown pigment released in oatmeal agar, glycerol-asparagine agar and inorganic salts-starch agar media, but these pigments were not produced in yeast-malt agar, peptone-yeast extract iron agar or tyrosine agar medium. These pigments are pH indicator that changes from faint brown by adding HCl (0.05 M) to colorless. The morphology of the strain NEAE-K was seen on inorganic salt-starch agar; there are no verticils or mycelium fragmentation. The chains of spore are spirals; spirals are open or closed and the surface of the spore is smooth (Fig. 4).

**Table 1:** Culture features of the *Streptomyces* sp. strain NEAE-K.

ISP Media	Growth	Diffusible pigment	Revese side of colony	Aerial mycelium
Yeast extract -malt extract agar (ISP-2)	Excellent	Non-pigmented	Brown	Gray
Oatmeal agar (ISP-3)	Excellent	Faint brown	Brown	Gray
Inorganic salt-starch agar (ISP-4)	Excellent	Faint brown	Brown	Gray
Glycerol-asparagines agar (ISP-5)	Excellent	Faint brown	Brown	Gray
Peptone-yeast extract iron agar (ISP-6)	Excellent	Non-pigmented	Faint brown	Gray
Tyrosine agar (ISP-7)	Very good	Non-pigmented	Faint brown	Whitish gray

### Physiological and chemotaxonomic features

Table 2 displays the physiological and morphological features of strain NEAE-K. D- xylose, D-galactose, cellulose, ribose, trehalose, rhamnose, maltose, L-arabinose, D-mannose, D-glucose, raffinose, D-fructose, and sucrose were used for growth. No antimicrobial activities have been reported against *Klebsiella*, *Bacillus subtilis*, *Pseudomonas aeruginosa*, *Escherichia coli*, *Staphylococcus aureus*, *Sacchromyces cerevisiae, Candida albicans*, *Fusarium oxysporum*, *Bipolaris oryzae*, *Aspergillus niger*, *Rhizoctonia solani* or *Alternaria solani*. Protease, α–amylase, uricase, cellulase and asparaginase were produced by the strain NEAE-K while lecithinase was not produced. Hydrogen sulphide production by the strain NEAE-K was negative while gelatin liquification, coagulation and peptonization of milk and reduction of nitrate to nitrite were positive. No melanoid pigments were formed. The strain NEAE-K showed the highest NaCl tolerance up to 7% (w/v). The optimum growth pH and temperature were 7 and 30 °C; respectively. The whole-cell hydrolyzates contained mainly galactose (28.41 mg/g), arabinose (7.46 mg/g) and fructose (9.13 mg/g). It has been evident that the strain NEAE-K belongs to *Streptomyces* genus, on the basis of the physiological, cultural, morphological and chemotaxonomic characteristics (Table 2).

**Table 2:** Phenotypic and physiological characteristics of *Streptomyces* sp. strain NEAE-K and its closest phylogenetic neighbors.

Characteristic	*Streptomyces* sp. strain NEAE-K	*Streptomyces rochei*	*Streptomyces olivaceus*	*Streptomyces plicatus*
Spore chain morphology	Spirals	Open spirals	Open spirals	Spirales
Spore shape	Oval to rectangular			
Spore surface	Smooth	Smooth to warty	Smooth	Smooth
Reverse side of colony *	Brown	No distinctive	Grayed yellow	No distinctive
Color of spores *	Gray	Gray	Gray	Gray or grayish yellowish pink
Production of diffusible pigment	Faint brown	No pigment	No pigment	No pigment
**Maximum NaCl tolerance (%, w/v)**	7	5	2.5	
**Melanin production on**				
Tyrosine agar	−	−	−	−
Peptone-yeast extract iron agar	−	−	−	−
Tryptone-yeast extract broth	−	−	−	−
**Carbon sources (1%,w/v) utilization**				
Trehalose	+			
Cellulose	+		−	
Raffinose	+	±	±	±
Rhamnose	+	+	+	+
Ribose	+			
D(+) Xylose	+	+	+	+
D(+) Mannose	+		−	
L-arabinose	+	+	+	+
D(+) Glucose	+	+	+	+
Sucrose	+	±	±	±
D(+) Galactose	+			
Maltose	+			
D(−) Fructose	+	+	+	+
**Enzymes**				
Gelatinase	+	+	+	
Reduction of nitrates to nitrite	+			
H_2_S production	−	−	−	

Abbreviations: Blank cells, no data available; −, Negative; +, Positive; ±, Doubtful.* on yeast extract -malt extract agar.

“lecithinase was not produced while α–amylase, protease, cellulase, uricase and asparaginase were produced by *Streptomyces* sp. strain NEAE-K. Antimicrobial activities were negative”. Coaggulation and peptonization of milk were positive.

### Phylogenetic analysis

**Figure 5:**
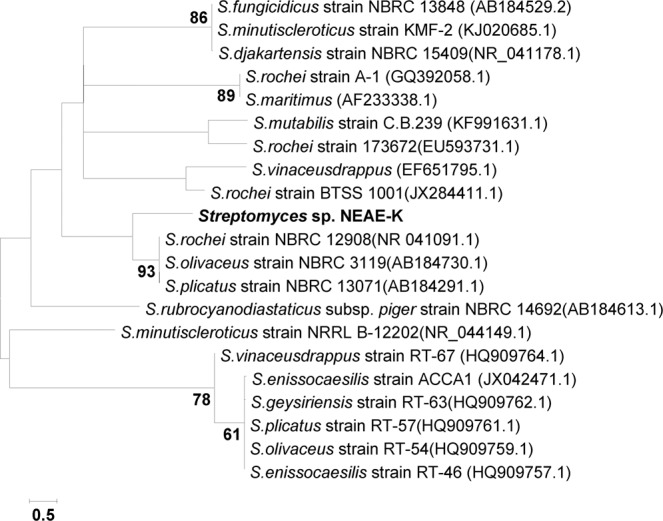
The neighbor-joining algorithm phylogenetic tree of strain NEAE-K and related species of the genus *Streptomyces*. “Only bootstrap values above 50%, expressed as percentages of 1000 replications, are shown at the branch points”.

The sequence of the 16 S rRNA gene for the NEAE-K strain has been estimated to be 1532 bp. The sequencing product has been deposited under the accession number KJ200343 in the database of the GenBank. The GenBank database search by BLAST^29^ revealed its similarity to the *Streptomyces* genus. A phylogenetic tree (Fig. 5) was constructed with MEGA software version 4.0^30^ using neighbor-joining algorithm method^31^. Phylogenetic tree shows that the NEAE-K strain joins a clad with *Streptomyces rochei* strain NBRC 12908 (NR_041091.1), *Streptomyces plicatus* strain NBRC 13071 (AB184291.1) and *Streptomyces olivaceus* strain NBRC 3119 (AB184730.1).

A comparison between NEAE-K strain and its closest phylogenetic neighbors (*Streptomyces rochei*, *Streptomyces olivaceus* and *Streptomyces plicatus*) showed significant variations (Table 2) in that it produced faint brown diffusible pigment and has maximum NaCl tolerance of 7% (w/v). Strain NEAE-K varied also in its pattern of utilization of some carbon sources from its closest phylogenetic neighbors. It has been clear that the NEAE-K strain is a new sub-species of *Streptomyces rochei* and the proposed name for this strain is *Streptomyces rochei* subsp. *chromatogenes* NEAE-K.

### Plackett–Burman design to identify significant factors affecting L-asparaginase production

Sixteen independent factors including A (incubation time), B (moisture content), C (inoculum size), D (pH), E (temperature), F (wheat bran + soybean meal; 1:1), G (dextrose), H (fructose), J (L-asparagine), K (K_2_HPO_4_), L (KNO_3_), M (yeast extract), N (MgSO_4_.7H_2_O), O (NaCl), P (FeSO_4_. 7H_2_O), Q (CaCl_2_) and three dummy factors were screened based on their impacts on the production of L-asparaginase using 20 trials of Plackett–Burman experimental design (Table 3). Table 3 presents the results of Plackett-Burman design under SSF conditions. The minimum activity of L-asparaginase (22.71 U/gds) was reached in run no. 18, while the maximum activity of L-asparaginase (140.27 U/gds) was achieved in run no. 9.

**Table 3:** Plackett–Burman experimental design under SSF conditions.

Std	Run	A	B	C	D	E	F	G	H	J	K	L	M	N	O	P	Q	L-asparaginase activity (U/gds)		Residuals
																		Actual	Predicted	
3	1	1	−1	1	1	−1	−1	1	1	1	1	−1	1	−1	1	−1	−1	73.03	73.47	−0.43
2	2	−1	1	1	−1	−1	1	1	1	1	−1	1	−1	1	−1	−1	−1	55.29	55.88	−0.60
16	3	1	1	1	1	−1	1	−1	1	−1	−1	−1	−1	1	1	−1	1	91.46	90.79	0.67
6	4	−1	−1	1	1	−1	1	1	−1	−1	1	1	1	1	−1	1	−1	87.03	83.72	3.30
8	5	−1	−1	−1	−1	1	1	−1	1	1	−1	−1	1	1	1	1	−1	126.62	125.75	0.86
18	6	−1	−1	1	1	1	1	−1	1	−1	1	−1	−1	−1	−1	1	1	124.91	128.21	−3.30
14	7	1	1	−1	1	−1	1	−1	−1	−1	−1	1	1	−1	1	1	−1	99.65	100.32	−0.67
12	8	−1	1	−1	1	−1	−1	−1	−1	1	1	−1	1	1	−1	−1	1	97.61	99.61	−2.01
1	9	1	1	−1	−1	1	1	1	1	−1	1	−1	1	−1	−1	−1	−1	140.27	142.54	−2.27
7	10	−1	−1	−1	1	1	−1	1	1	−1	−1	1	1	1	1	−1	1	101.36	102.15	−0.79
10	11	−1	1	−1	−1	−1	−1	1	1	−1	1	1	−1	−1	1	1	1	106.14	102.71	3.43
9	12	1	−1	−1	−1	−1	1	1	−1	1	1	−1	−1	1	1	1	1	87.37	90.79	−3.43
13	13	1	−1	1	−1	1	−1	−1	−1	−1	1	1	−1	1	1	−1	−1	110.23	109.61	0.63
15	14	1	1	1	−1	1	−1	1	−1	−1	−1	−1	1	1	−1	1	1	106.14	106.50	−0.36
19	15	1	−1	−1	1	1	1	1	−1	1	−1	1	−1	−1	−1	−1	1	77.81	74.39	3.43
20	16	−1	−1	−1	−1	−1	−1	−1	−1	−1	−1	−1	−1	−1	−1	−1	−1	93.17	93.80	−0.63
5	17	−1	1	1	−1	1	1	−1	−1	1	1	1	1	−1	1	−1	1	115.69	113.69	2.01
17	18	−1	1	1	1	1	−1	1	−1	1	−1	−1	−1	−1	1	1	−1	22.71	24.98	−2.27
4	19	1	1	−1	1	1	−1	−1	1	1	1	1	−1	1	−1	1	−1	113.71	111.63	2.08
11	20	1	−1	1	−1	−1	−1	−1	1	1	−1	1	1	−1	−1	1	1	122.71	122.34	0.36
**Variable level**		**Days**	**%**	**%; v/v**	**pH**	**°C**	**g,1:1**	**g/L**	**g/L**	**g/L**	**g/L**	**g/L**	**g/L**	**g/L**	**g/L**	**g/L**	**g/L**			
−1		5	50	2	6.5	30	10	2	2	7	1	0.5	1	0.1	0.1	0.01	0.01			
1		7	70	4	8.5	37	15	4	4	10	2	1	4	0.5	0.5	0.02	0.02			

The independent factors are: A (incubation time; days), B (moisture content; %), C (inoculum size; %), D (pH), E (temperature; °C), F (wheat bran + soybean meal; 1:1 g), G (dextrose; g/L), H (fructose; g/L), J (L-asparagine; g/L), K (K2HPO4; g/L), L (KNO3; g/L), M (yeast extract; g/L), N (MgSO4.7H2O; g/L), O (NaCl; g/L), P (FeSO4. 7H2O; g/L) and Q (CaCl2; g/L).

The relationship between L-asparaginase production and the independent factors is determined by the statistical analysis of the Plackett-Burman design results (Table 4). The interpretation of the data was based on the signs of the coefficients and main effects (positive or negative effects on L-asparaginase production). On the basis of the calculated main effects of the factors (Table 4), ten of the sixteen factors affect the production of L-asparaginase positively; they are yeast extract, fructose, soybean meal + wheat bran, temperature, incubation time, MgSO_4_.7H_2_O, K_2_HPO_4_, KNO_3_, FeSO_4_. 7H_2_O and CaCl_2_; while the other six factors (pH, dextrose, moisture content, inoculum size, L-asparagine and NaCl) exerted negative effects on the production of L-asparaginase.

**Table 4:** Plackett-Burman design statistical analysis.

Term	Coefficient estimate	Main Effect	Contribution (%)	*F*-value	*P*-value	Confidence level
Intercept	97.65			55.69	0.0178*	98.22
A-Incubation time	4.59	9.19	3.10	24.31	0.0388*	96.12
B-Moisture content	−2.78	−5.56	1.13	8.89	0.0964	90.36
C-Inoculum size	−6.72	−13.45	6.64	52.11	0.0187*	98.13
D- pH	−8.72	−17.43	11.16	87.54	0.0112*	98.88
E- Temperature	6.30	12.60	5.83	45.73	0.0212*	97.88
F-Soybean meal + wheat bran	2.96	5.93	1.29	10.12	0.0862	91.38
G-Dextrose	−11.93	−23.86	20.91	164.01	0.0060*	99.4
H-Fructose	7.90	15.81	9.18	71.97	0.0136*	98.64
J-L-asparagine	−8.39	−16.78	10.34	81.12	0.0121*	98.79
K- K_2_HPO_4_	7.95	15.91	9.29	72.88	0.0134*	98.66
L-KNO_3_	1.32	2.64	0.25	9.00	0.0955	90.45
M-Yeast extract	9.37	18.73	12.88	101.05	0.0098*	99.02
N-MgSO_4_.7H_2_O	0.035	0.07	0.00	0.006	0.9430	5.7
O-NaCl	−4.22	−8.44	2.61	20.50	0.0455*	95.45
P-FeSO_4_. 7H_2_O	2.05	4.11	0.62	4.85	0.1584	84.16
Q-CaCl_2_	5.47	10.95	4.40	34.53	0.0278*	97.22
Std. Dev.	4.17	**R**^2^	0.9974			
Mean	97.65	**Adj R**^2^	0.9795			
**C.V.%**	4.27	**Pred R**^2^	0.9014			
**PRESS**	1337.59	**Adeq Precision**	32.58			

^*^Significant values.

When the determination coefficient (R^2^) value is closer to 1, the design used is better and stronger for predicting the response^32^. The value of R^2^ in this study is 0.9974, indicating the fitness of the model. In addition, the value of the adjusted R^2^ of 0.9795 is also very high, implying that the model has a great significance. A higher predicted R^2^ (0.9014) implying that this model is adequate for predicting the value of L-asparaginase production in the range of factors used.

In order to assess the significance of the variables, both *P* and *F*-values were calculated (Table 4). In the current experiment, variables evidencing *P*- values of less than or equal to 0.05 (confidence levels of more than or equal to 95%) were considered to have significant impacts on L-asparaginase production. The most significant factor was dextrose, with a confidence level of 99.4, *P*-value of 0.0060 and *F*-value of 164.01, followed by yeast extract (confidence level 99.02, *F*-value 101.05 and *P*-value 0.0098), pH (confidence level 98.88, *F*-value 87.54 and *P*- value 0.0112). The *F*-value of the model of 55.69 (Table 4) meaning the model’s significance. The *P*-value of 0.0178 (<0.05) confirms significant model terms. The equation of the model fitted with a regression analysis is given by ignoring the independent variables that were insignificant (*P* > 0.05):

1 $${\rm{Y}}=97.65+4.59\,{\rm{A}}-6.72\,{\rm{C}}-8.72\,{\rm{D}}+6.3\,{\rm{E}}-11.93\,{\rm{G}}+7.9\,{\rm{H}}-8.39\,{\rm{J}}+7.95\,{\rm{K}}+9.37\,{\rm{M}}-4.22\,{\rm{O}}+5.47\,{\rm{Q}}$$

where the L-asparaginase production is Y and the independent factors are: A (incubation time), C (inoculum size), D (pH), E (temperature), G (dextrose), H (fructose), J (L-asparagine), K (K_2_HPO_4_), M (yeast extract), O (NaCl) and Q (CaCl_2_).

**Figure 6:**
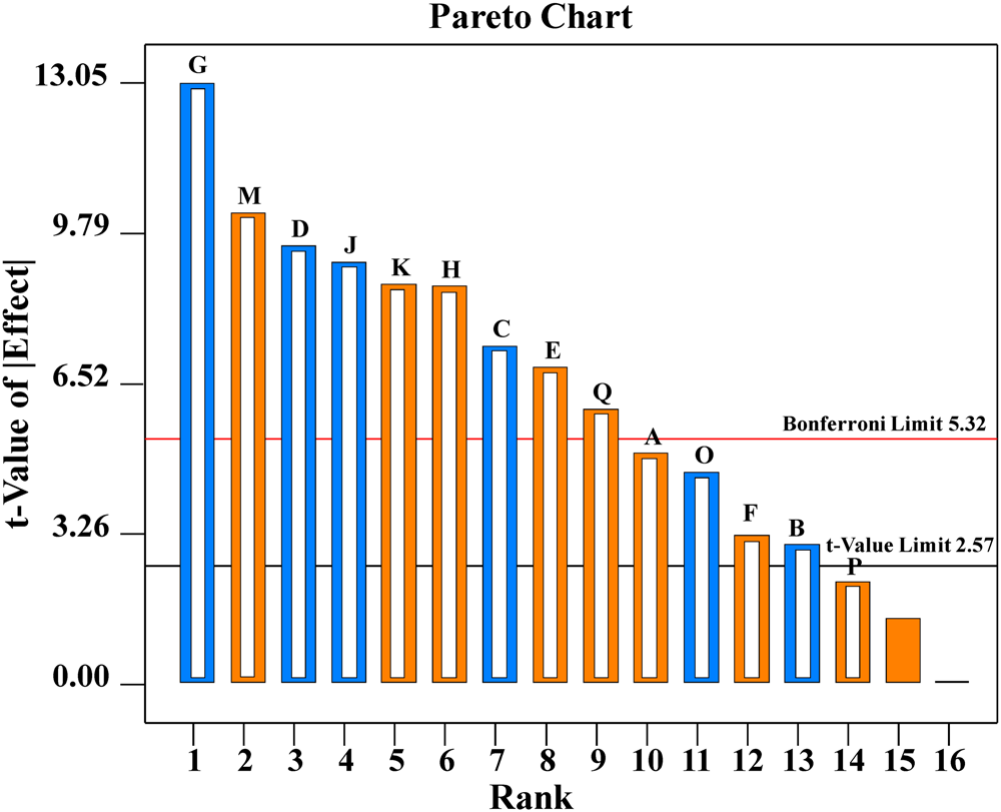
The Pareto chart shows the order of significance of the variables affecting L-asparaginase production.

The significance order of the factors influencing the production of L-asparaginase illustrated in the Pareto chart (Fig. 6). Among the variables screened; G (dextrose), M (yeast extract), and D (pH) have been defined as the most significant factors for the production of L-asparaginase and chosen for central composite design optimization.

To evaluate the precision of Plackett-Burman design, the medium of the following composition was used in the confirmatory experiment (g/L): L-asparagine 7, yeast extract 4, Wheat bran + soybean meal 15, fructose 4, dextrose 2, FeSO_4_. 7H_2_O 0.02, CaCl_2_ 0.01, NaCl 0.1, MgSO_4_.7H_2_O 0.5, K_2_HPO_4_ 2, KNO_3_ 1. The moisture content was 50% and pH 6.5. Two mL of the inoculum was used for medium inoculation and the inoculated flasks were incubated at a temperature of 37°C for 7 days. The obtained maximum production of L-asparaginase was 145.4 U/gds, which was increased 3.24 times compared with production of L-asparaginase achieved prior to the application of Plackett-Burman design (44.821 U/gds). Singh and Srivastava^33^ reported that the optimization of the solid state fermentation media and parameters resulted in a 14% increase in the L-asparaginase activity.

### Optimization of L-asparaginase production by central composite design (CCD)

Central composite design of response surface methodology (RSM) is an effective experimental technique designed for optimizing the production conditions. The main advantage of RSM is that it requires little experimental runs for multiple parameters to provide sufficient information for statistically acceptable results. It is faster, reduces the study’s time and effort and avoids misinterpretation that occurs with the conventional approach. The regression analysis of the RSM helps in identifying the significant factors, exploring their relationships and predict the response^34^.

The optimal levels of the three variables; pH (X_1_), dextrose (X_2_) and yeast extract (X_3_) and their mutual effects on L-asparaginase production were estimated by CCD. Table 5, includes the design matrix for 20 experimental runs, the central point was repeated six times (2, 5, 7, 8, 14, and 20). Table 5 also presents different combinations of the three independent factors and L-asparaginase production (predicted and observed values).

**Table 5:** Central composite design for L-asparaginase production as influenced by pH (X_1_), dextrose (X_2_) and yeast extract (X_3_).

Std	Run	Factors			L-asparaginase production (U/gds)		Residuals
		X_1_	X_2_	X_3_	Experimental	Predicted	
14	1	0	0	1.68	88.60	89.22	−0.63
19	2	0	0	0	177.64	169.93	7.70
12	3	0	1.68	0	127.17	128.23	−1.06
4	4	1	1	−1	89.66	90.61	−0.95
16	5	0	0	0	173.70	169.93	3.76
10	6	1.68	0	0	93.88	93.60	0.28
18	7	0	0	0	166.37	169.93	−3.56
15	8	0	0	0	163.30	169.93	−6.63
3	9	−1	1	−1	129.68	125.75	3.93
11	10	0	−1.68	0	112.36	114.99	−2.63
8	11	1	1	1	99.60	98.48	1.12
6	12	1	−1	1	88.36	89.68	−1.32
5	13	−1	−1	1	107.66	104.09	3.57
20	14	0	0	0	169.96	169.93	0.02
2	15	1	−1	−1	97.31	94.51	2.80
13	16	0	0	−1.68	91.90	94.97	−3.07
9	17	−1.68	0	0	131.29	135.27	−3.98
7	18	−1	1	1	123.55	123.74	−0.19
1	19	−1	−1	−1	120.30	118.80	1.50
17	20	0	0	0	169.27	169.93	−0.67
**Level**	**pH**	**Dextrose (g/L)**	**Yeast extract (g/L)**				
−1.68	5.32	1.66	1.32				
−1	6	2	2				
0	7	2.5	3				
1	8	3	4				
1.68	8.68	3.34	4.68				

Based on the levels of the three independent variables, the results revealed considerable variability in the L-asparaginase production. The maximum L-asparaginase production was obtained in the central run no. 2 with a value of 177.64 U/gds where pH was 7, yeast extract was 3 g/L and dextrose was 2.5 g/L. While the minimum L-asparaginase production (88.36 U/gds) has been obtained in the central run no. 12, where pH was 8, yeast extract was 4 g/L and dextrose was 2 g/L.

### Statistical analysis of the data

Table 6 presents the statistical analysis of the data, coefficients, *P-*value and *F*-value. The model determination coefficient (R^2^) of 0.9898 means that the model could explain 98.98 percent of the variance in the production of L-asparaginase. Data were interpreted based on the significance (*P* < 0.05). Statistical analysis of the data shows that the model is very significant, as demonstrated by the Fisher’s *F-*value (107.65) and a very low *P*-value (0.0001 < 0.05). It is evident from the significance values (Table 6) that the linear coefficients of pH (X_1_), dextrose (X_2_) and quadratic effects of pH (X_1_), dextrose (X_2_) and yeast extract (X_3_) are significant. The *P-*values indicate that the linear coefficient of yeast extract (X_3_) is not significant (*P-*value = 0.1947). On the other hand, the different interactions between the three variables studied (X_1_, X_2_; X_1_ X_3_ and X_2_ X_3_) are not significant (*P*-values of 0.1225, 1552, 0.0763; respectively), indicating that they have not significantly contributed to the enhancement of L-asparaginase production.

**Table 6:** Statistical analysis for CCD results of L-asparaginase production.

Source of variance		Sum of Squares	*df*	Mean Square	*F-*value	*P-*value *P*rob > *F*	Coefficient estimate
Model		20014.73	9	2223.86	107.65	<0.0001*	169.93
Linear effects	X_1_ - pH	2095.56	1	2095.56	101.44	<0.0001*	−12.39
	X_2_- Dextrose	211.67	1	211.67	10.25	0.0095*	3.94
	X_3_ -Yeast extract	39.90	1	39.90	1.93	0.1947	−1.71
Interaction effects	X_1_ X_2_	58.77	1	58.77	2.85	0.1225	−2.71
	X_1_ X_3_	48.83	1	48.83	2.36	0.1552	2.47
	X_2_ X_3_	80.69	1	80.69	3.91	0.0763	3.18
Quadratic effects	X_1_^2^	5548.66	1	5548.66	268.60	<0.0001*	-19.62
	X_2_^2^	4206.03	1	4206.03	203.60	<0.0001*	-17.08
	X_3_^2^	10913.49	1	10913.49	528.30	<0.0001*	-27.52
Error effects	Lack of Fit	76.07	5	15.21	0.58	0.7160	
	Pure Error	130.51	5	26.10			
**Std. Dev**.		4.55	**R**^**2**^			0.9898	
**Mean**		126.08	**Adj R**^**2**^			0.9806	
**C.V.%**		3.61	**Pred R**^**2**^			0.9607	
**PRESS**		794.12	**Adeq Precision**			25.11	

Table 7 shows the fit summary results, the quadratic model is a highly significant and adequate model fitting L-asparaginase production with a very low probability value (*P-*value < 0.0001) and non-significant lack of fit (*P-*value = 0.7160, *F*-value = 0.58). The quadratic model summary statistics has the largest adjusted (0.9806) and predicted R^2^ (0.9607) and lowest standard deviation (4.55).

**Table 7:** Fit summary for the results of CCD of L-asparaginase production.

Model Summary Statistics					
Source	Predicted R^2^	R^2^	Adjusted R^2^	SD	PRESS
Linear	−0.2032	0.1161	−0.0497	33.42	24329.46
2FI	−1.0633	0.1254	−0.2783	36.88	41723.40
Quadratic	0.9607	0.9898	0.9806	4.55	794.12
**Lack of Fit Tests**					
**Source**	***Df***	***SS***	***P-*value**	***F-*value**	***MS***
Linear	11	17743.66	0.0001*	61.80	1613.06
2FI	8	17555.37	<0.0001*	84.07	2194.42
Quadratic	5	76.07	0.7160	0.58	15.21
Pure Error	5	130.51			26.10
**Sequential Model Sum of Squares**					
**Source**	***Df***	***SS***	***P-*value**	***F-*value**	***MS***
Linear vs Mean	3	2347.14	0.5655	0.70	782.38
2FI vs Linear	3	188.29	0.9862	0.05	62.76
Quadratic vs 2FI	3	17479.30	<0.0001*	282.04	5826.43
Residual	6	164.73			27.45

To evaluate the correlation between different factors and to calculate the highest production of L-asparaginase corresponding to the optimal values of pH, dextrose and yeast extract, the equation of the second-order polynomial model (Eq. 2) has been suggested. Depending on the optimum levels of the independent variables (X_1_, X_2_ and X_3_), the maximum L-asparaginase production is predictable:

2 $${{\rm{Y}}}_{({\rm{L}}-{\rm{asparaginase}}{\rm{production}})}=169.93-12.39{{\rm{X}}}_{1}+3.94{{\rm{X}}}_{2}-1.71{{\rm{X}}}_{3}-2.71{{\rm{X}}}_{1}{{\rm{X}}}_{2}+2.47{{\rm{X}}}_{1}{{\rm{X}}}_{3}+3.18{{\rm{X}}}_{2}{{\rm{X}}}_{3}-19.62{{{\rm{X}}}_{1}}^{2}-17.08{{{\rm{X}}}_{2}}^{2}-27.52{{{\rm{X}}}_{3}}^{2}$$

where Y is the value of predicted production of L-asparaginase, X_1_ is the coded value of pH, dextrose (X_2_) and yeast extract (X_3_).

### Model accuracy checking

**Figure 7:**
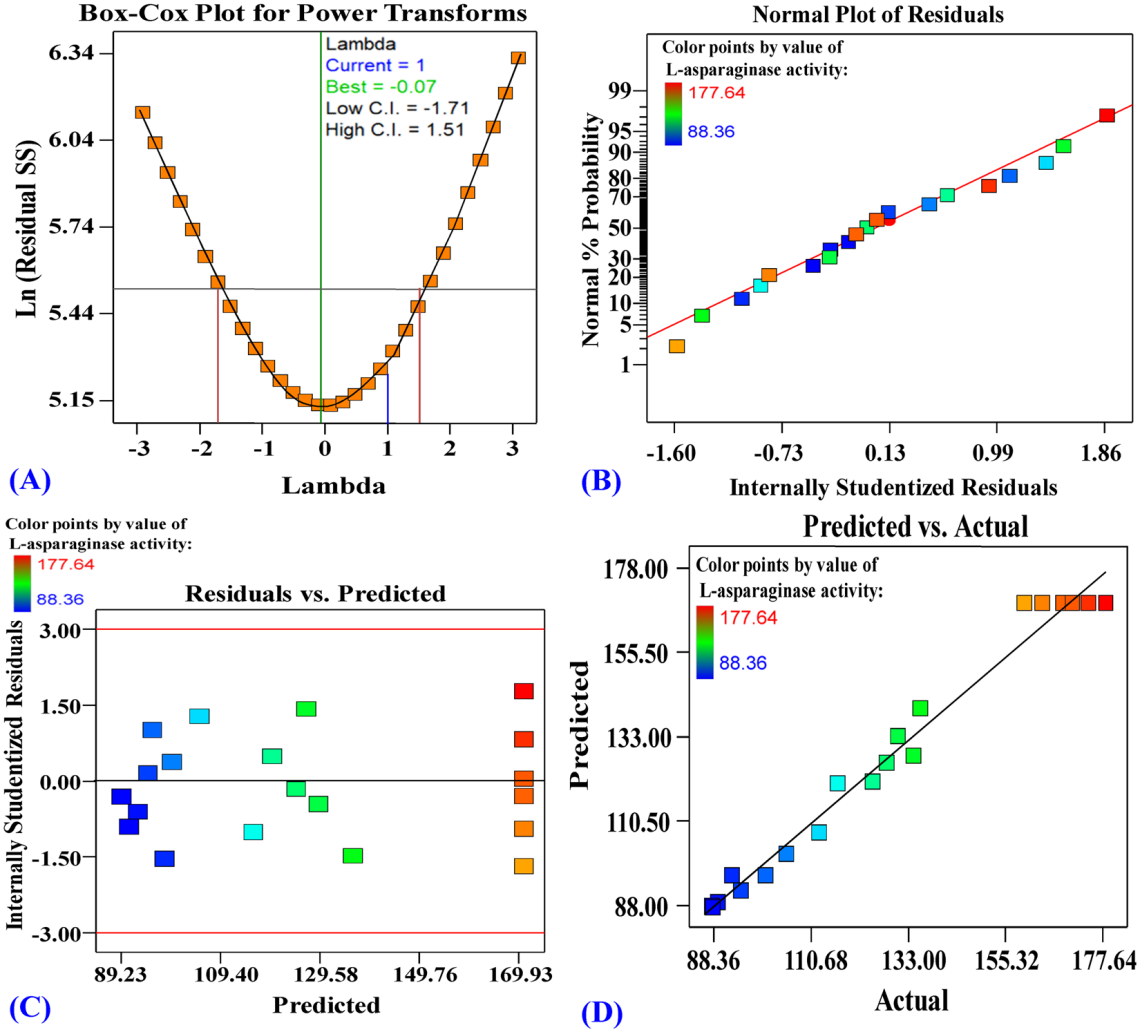
(**A**) Box- Cox plot of model transformation, (**B)** Normal probability plot of internally studentized residuals (**C**) plot of internally studentized residuals versus predicted values and (**D**) plot of predicted versus actual values of L-asparaginase production.

Some statistics were carried out for confirmation of the design’s accuracy. Figure 7A shows, the Box–Cox graph, the green line represents the best lambda value (Lambda = −0.07) and the blue line represents the current transformation (Lambda = 1). While the red lines represent the lowest and the highest values of confidence intervals between −1.71 and 1.51. As Lambda’s best value lies between the minimum and maximum confidence intervals, no transformations of the data were recommended. The blue line lies between the two red lines; it implies that the model is well fitting to the experimental data obtained. Figure 7B displays NPP (the normal probability plot) of the residuals. The residuals have been drawn versus the normal scores which are the cumulative probability. The resulting points are shown in the NPP of the residues close the diagonal line in such a way that they are normally distributed, indicating a good model fit. Figure 7C shows a plot of the predicted L-asparaginase production against the residuals. The residuals scattered uniformly and randomly above and below the center line of zero with no obvious pattern, indicating that the residuals have a constant variance, confirming the model’s adequacy. Figure 7D shows a plot of predicted versus the actual L-asparaginase production, showing the points close to the fitted line, supporting a significant correlation between the predicted L-asparaginase production of the model and its actual results.

### Three-dimensional surface plots

**Figure 8:**
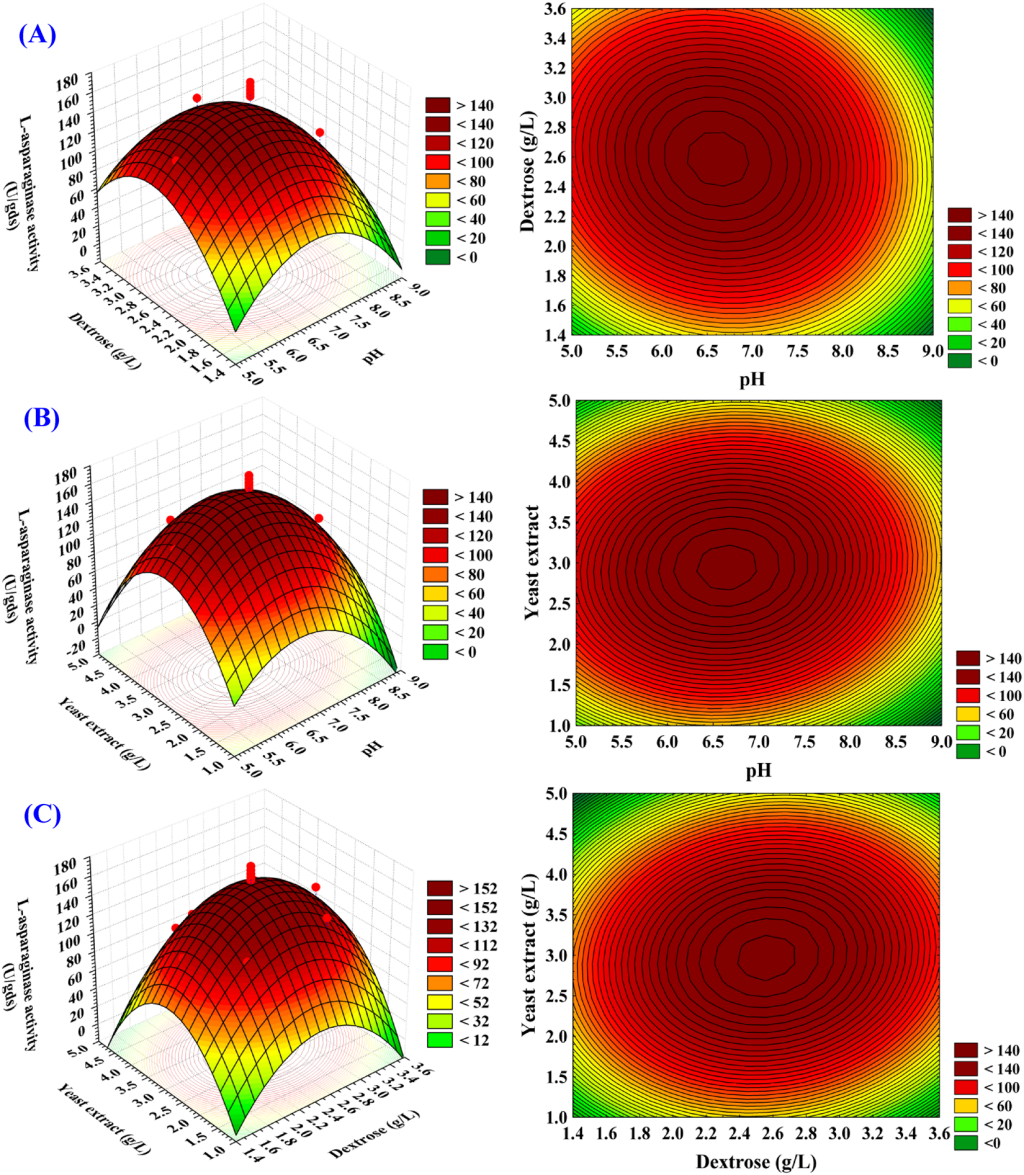
(**A–C**). 3D plots showing the mutual effects of pH (X_1_), dextrose (X_2_) and yeast extract (X_3_) on the production of L-asparaginase.

The three-dimensional surface plots were designed to identify the optimal concentrations and the interaction between the factors for maximal L-asparaginase production. L-asparaginase production was plotted on the Z-axis against two factors, while the third factor was set at its zero level. (Fig. 8A–C). Figure 8A illustrates the production of L-asparaginase as a response of pH (X_1_), dextrose (X_2_) by keeping yeast extract (X_3_) at zero level. It has been shown that the highest and lowest levels of dextrose were accompanied by low L-asparaginase production. At the moderate dextrose level, maximum L-asparaginase production was obtained. L-asparaginase production was maximized at the mid-pH levels. By solving the Eq. (2), the highest production of 172.15 U/gds L-asparaginase could be gained by using 3 g/L yeast extract at the optimal predicted concentration of dextrose (2.6 g/L) and pH (6.7).

The production of L-asparaginase by different microorganisms strongly depends on the medium pH. The diffusion of different components throughout the cell membrane and regulation of the metabolic processes in the cells are fundamentally affected by the pH^35^. Dharmaraj^36^ reported that the highest *Streptomyces noursei* MTCC 10469 production of L-asparaginase was achieved at pH 8. While, the maximum *Serratia marcescens* (NCIM 2919) L- asparaginase production was obtained at pH 7.4^37^. Varalakshmi and Raju^38^ recorded *Aspergillus terreus* MTCC 1782 highest production of L-asparaginase (191.3 U/gds) was achieved at pH 8 under SSF conditions.

Carbon sources are used in the formulations of microbial fermentation media to enhance the growth of the desired microorganism and subsequently resulted in a higher synthesis of primary metabolites (such as the enzymes) during fermentation process^39^. Carbon source acts as a major nutrient for cell proliferation and for production of L-asparaginase. Glucose, sucrose and fructose are found to be promising carbon sources for production of L-asparaginase^40^. The influence of carbon source on microbial growth and production of metabolites depends on several factors, including the carbon concentration^41^.

The role of glucose (D-glucose is also known as dextrose) in L-asparaginase synthesis is controversial. In general, glucose at higher concentrations was seen as a catabolic repressor in the bacterial L-asparaginase synthesis by *Erwinia aeroideae* and *Escherichia coli*^42,43^. This may be due to the suppression of catabolites of lactate transport components that stimulated synthesis of L-asparaginase^44^. The maximum production of L-asparaginase by *Escherichia coli* was achieved by using 0.25% glucose supplementation^45^. In contrast, the highest synthesis of L-asparaginase by *Bacillus circulans* was achieved when the strain was grown in the presence of 1-1.5 g of glucose supplementation in the fermentation medium^18^. *Serratia marcescens*^46^, *Bacillus* sp. WF67^47^ and *Pectobacterium carotovorum* MTCC 1428^48^ produce L- asparaginase maximally when glucose was used as a source of carbon. Glucose and starch were stated to be good sources of carbon for the highest production of L-asparaginase by *Streptomyces ginsengisoli*^49^. Dextrose (glucose) has been identified as the best carbon source at a concentration of 3 g/L having a positive influence on the production of *Streptomyces olivaceus* NEAE-119 glutaminase free L-asparaginase^50^.

Literature on fungal L-asparaginase production reported that *Fusaium oxysporum* had the highest L-asparaginase production with 0.3 percent glucose as a carbon source^51^. Maximum L-asparaginase production by *Bipolaris* sp. BR438 was obtained using the modified Czapek-Dox medium supplemented with 0.4% glucose^52^. *Aspergillus terrus* MTCC 1782, grown in the modified Czapek-Dox medium complemented by 0.6% soybean flour and 0.2% glucose, achieved the highest L-asparaginase synthesis^53^.

Figure 8B illustrates L-asparaginase production as a response of pH (X_1_), yeast extract (X_3_) by keeping dextrose (X_2_) at zero level. At the moderate levels of both pH and yeast extract, maximum L-asparaginase production has been obtained and a gradual decline in the production of L-asparaginase has been observed at higher levels of both pH and yeast extract. By analyzing Fig. 8B and predicting Eq. (2), the L-asparaginase production of 171.94 U/gds could be achieved with 2.5 g/L dextrose at the optimum predicted levels of pH (6.7) and 2.95 g/L yeast extract.

L-asparaginase production increases with supplementation of nitrogen sources, indicating that it is regulated by nitrogen; therefore it requires supplementary nitrogen sources in addition to the carbon source to enhance L-asparaginase production. Many microorganisms consume both organic and inorganic sources of nitrogen to generate components of the cell wall, proteins, nucleic acids and amino acids, industrial enzymes. Di-ammonium hydrogen phosphate (0.16%) was identified as a good source of nitrogen for L-asparaginase production by *Enterobacter aerogenes*^54^. As reported by Pallem *et al*.^51^, malt extract was the preferred source of nitrogen for maximum L-asparaginase production by *Fusarium oxysporum*. On the other hand, yeast extract was found to be a good source of nitrogen for the production of L-asparaginase by *Amycolatopsis* CMU-H002 (1.5%)^55^, *Streptomyces albidoflavus* (2%)^13^, *Erwinia aroideae*^56^ and *Erwinia carotovora* EC-113^57^. Liu and Zajic^56^ reported that *Erwinia aroideae* production of L-asparaginase was dependent on the concentration of yeast extract and the optimal concentration of 1.5 percent gave the maximum L-asparaginase. In addition, Verma *et al*^3^. indicated that the yeast extract was essential for cell growth as well as L-asparaginase production, which was inhibited at high concentrations of yeast extract^42,53^.

Figure 8C illustrates L-asparaginase production as a response of dextrose (X_2_), yeast extract (X_3_) by keeping pH (X_1_) at zero level. It has been shown that the larger production of L-asparaginase has been achieved at the middle level of dextrose (X_2_), yeast extract (X_3_) and the higher levels resulted in a decline in L-asparaginase production. By analyzing Fig. 8C and predicting Eq. (2), the maximal L-asparaginase production of 170.18 U/gds could be achieved with pH (7) and the optimal predicted levels of yeast extract concentration (2.97 g/L) and 2.55 g/L dextrose.

### Verification of the experimental model

The validation of the experimental results of *Streptomyces rochei* subsp. *chromatogenes* NEAE-K L-asparaginase production has been evaluated under optimum conditions from the CCD and compared to the predicted data. The highest predicted L-asparaginase production (172.15 U/gds) could be gained by using 3 g/L yeast extract, 2.6 g/L dextrose and pH 6.7. The experimental L-asparaginase activity (175.96 U/gds) was very close to that predicted by the regression model (172.15 U/gds), which confirmed the model’s validity.

Finally, the maximum *Streptomyces rochei* subsp. *chromatogenes* NEAE-K L-asparaginase production was found to be 175.96 U/gds by using the following medium compositions (g/L): L-asparagine 7, yeast extract 3, wheat bran + soybean meal 15, fructose 4, dextrose 2.6, CaCl_2_ 0.01, FeSO_4_. 7H_2_O 0.02, K_2_HPO_4_ 2, NaCl 0.1, KNO_3_ 1, MgSO_4_.7H_2_O 0.5. The moisture content was 50% and pH 6.7. The medium was inoculated with 2 mL of the inoculum. The inoculated medium was incubated at a temperature of 37°C for 7 days.

El-Naggar *et al*.^26^ used a central composite design based optimization to investigate and analyze the combined effect of three variables that were significant for *Streptomyces brollosae* NEAE-115 L-asparaginase production. The highest production of L-asparaginase was 149.65 U/gds at the optimal levels of K_2_HPO_4_ (2.5 g/L), L-asparagine (12 g/L) and soybean + wheat bran (16 g/L). The optimized levels of process variables for maximum experimental L-asparaginase production (3.74 U) by *Cladosporium* sp. were found to be incubation temperature (30 °C), pH (5.8) and moisture content (58%) using wheat bran as substrate^58^. Ghosh *et al*.^37^ used traditional one variable at a time method and Box-Behnken design to evaluate the effect of different nutritional ingredients and cultural conditions on *Serratia marcescens* L-asparaginase production. The highest production of L-asparaginase (5.86 U/gds) using Box-Behnken design based optimization was obtained by using the optimal levels of the most significant variables: initial substrate moisture content 50%, pH 7.4, temperature 35.5°Cand 7.5 g of coconut oil cake. On the other hand, Kumar *et al*.^59^ found that the maximum production of L-asparaginase by *Serratia marcescens* was 83.16 U/g under the optimum conditions of time (48 h), temperature (28°C), pH (7.5) and moisture content (60%) when the fermentation medium supplemented with 0.6% L-asparagine and 1.5% sucrose. Furthermore, Hosamani and Kaliwal^60^ found that, maximum production of *Fusarium equiseti* L-asparaginase was 8.51 U which obtained under the following conditions: yeast extract (0.5%, w/v), glucose (0.5%, w/v), inoculum volume (20%, v/w), particle size (3 mm), initial moisture content (70%, v/w), ammonium sulphate (0.5%, w/v) and incubation period (48 h). Singh and Srivastava^33^ found that the maximum production of *Bacillus aryabhattai* strain ITBHU02 L-asparaginase was 16.1 U/gds and obtained on wheat bran supplemented with 0.25% (w/w) L-asparagine and 1% (w/w) yeast extract. The moisture content was 100% (v/w) and pH 7.5. The inoculated medium had been incubated for 36 hours at a temperature of 30 °C.

### L-asparaginase purification

**Figure 9:**
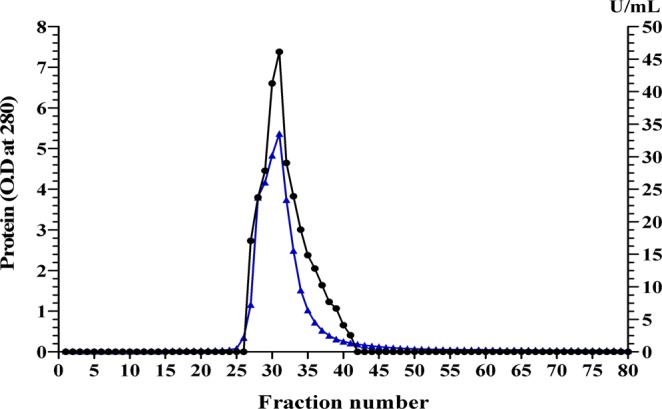
Purification of L-asparaginase produced by *Streptomyces rochei* subsp. *chromatogenes* NEAE-K using ion exchange on DEAE Sepharose CL-6B. (▴) refer to protein, (•) refer to L-asparaginase activity.

For purification of L-asparaginase, crude filtrate of culture with overall activity 24410.1 U, protein content 3304 mg and specific activity of 7.39 U/mg proteins was used. The ammonium sulfate precipitation partially purified L-asparaginase and the dialyzed enzyme had 226.8 mg protein content, 32.37 U/mg protein specific activity and enzyme recovery of 30.07% with 4.38 purification fold (Table 8). The purified enzyme was obtained with the packed column of DEAE Sepharose CL-6B, resulting in 80 fractions with a single peak (Fig. 9). The purified L-asparaginase collected from the packed column of DEAE Sepharose CL-6B has 4.8 mg protein content with overall activity 573.41 and the specific activity of 119.51 U/mg protein with 16.18 purification folds. Sahu *et al*.^61^ reported the purification of L-asparaginase produced by an actinomycete strain LA-29 with 1.9% protein recovery and 13.57 U/mg protein specific activity and 18 purification fold.

**Table 8:** Purification steps summary.

Purification step	Total protein content (mg)	L-asparaginase activity			
		Total activity (U)	Specific activity (U/mg protein)	Recovery (%)	Purification fold
Culture filtrate	3304	24410.1	7.39	100	—
(NH_4_)_2_SO_4_, post dialysis	226.8	7340.43	32.37	30.07	4.38
Ion exchange on DEAE Sepharose CL-6B	4.80	573.41	119.51	2.35	16.18

### SDS-PAGE and determination of monomeric molecular weight of the protein

**Figure 10:**
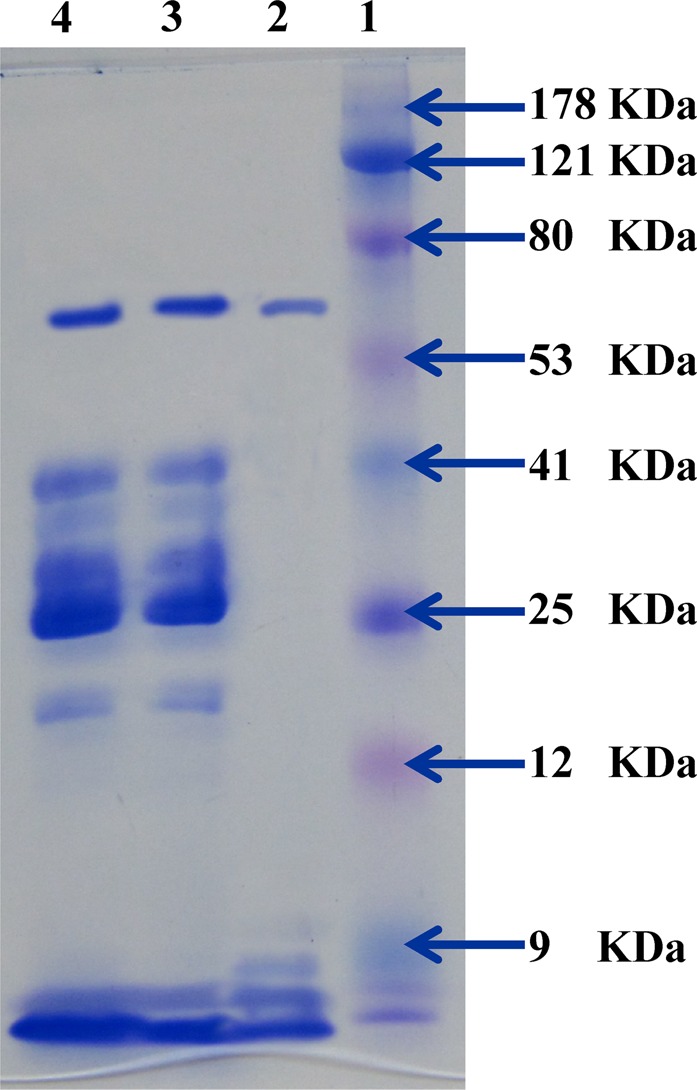
SDS-polyacrylamide gel electrophoresis of the purified L-asparaginase produced by *Streptomyces rochei* subsp. *chromatogenes* NEAE-K. Lane 1: Protein marker; Lane 2: Purified L-asparaginase; Lanes 3, 4: Ammonium sulphate fractions.

Protein separation by SDS–PAGE method was used to determine the monomeric molecular weight of L-asparaginase^62^. Only one protein band has been resolved with a molecular mass of 64 kDa (Fig. 10). L-asparaginase molecular weight was shown to be different depending on the source of the enzyme. Purified L-asparaginase from *Streptomyces gulbargensis*^63^, *Streptomyces fradiae* NEAE-82^64^, *Streptomyces* PDK2^65^, *Streptomyces noursei*^36^ and *Streptomyces albidoflavus*^13^ exhibited a molecular weight of 85, 53, 140, 102 and 112 kDa; respectively.

### Cytotoxic activity of L-asparaginase against human cancer cell lines

**Figure 11:**
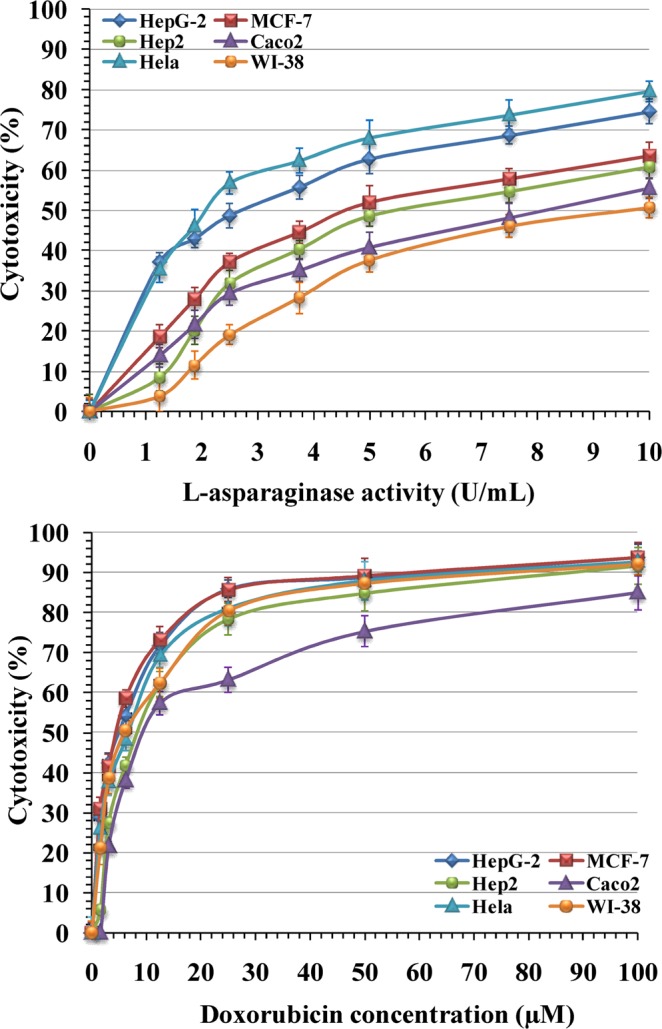
The anticancer effects of (**A**) the purified L-asparaginase, and (**B**) doxorubicin on HepG-2, MCF-7, Hep2, Caco-2, Hela and WI-38 cells after 24 h of incubation.

The purified enzyme has been assessed on five human cancer cell lines for its *in vitro* anticancer effect via the standard MTT assay. The cancer cell lines, namely; epitheliod carcinoma (HeLa), Hep2 (epidermoid larynx carcinoma), MCF-7 (breast carcinoma), Caco2 (Colorectal adenocarcinoma), and hepatocellular carcinoma (HepG-2). Human lung fibroblast, WI-38 (normal cell line) was also used. The results showed that L-asparaginase displayed varying degrees of inhibitory activity against the tested human cell lines of the tumors and normal cell line (Fig. 11).

L-asparaginase at 10 U/mL inhibited the cell viability after 24 hrs of incubation of HeLa, HepG-2, MCF-7, Hep2, Caco2 and normal WI-38 cell lines by 79.6, 74.6, 63.5, 60.8, 55.5 and 50.7%; respectively. The treatment IC_50_ on the tested human cell lines ranged from 2.16 ± 0.2 to 8.5 ± 0.5 U/mL (Table 9). The very strong cytotoxic activity displayed by L-asparaginase was against HeLa and HepG-2 cell lines with an IC_50_ of 2.16 ± 0.2 and 2.54 ± 0.3 U/mL; respectively. A moderate cytotoxic activity was also demonstrated by L-asparaginase against MCF-7, Hep2 and Caco2 cell lines with an IC_50_ of 4.95 ± 0.4, 5.90 ± 0.4 and 7.59 ± 0.5; respectively. Weak cytotoxic activity was also demonstrated by L-asparaginase against the normal cell line (WI-38) with an IC_50_ of 8.5 ± 0.5.

**Table 9:** Cytotoxicity IC_50_ and selectivity index (SI) of DOX and the purified L-asparaginase agaist different cell lines.

Compounds	HeLa	HepG-2	MCF-7	Hep2	Caco2	WI-38
**DOX (µM)**						
Cytotoxicity IC_50_ (μM)	5.57 ± 0.3	4.50 ± 0.2	4.17 ± 0.2	8.54 ± 0.6	12.49 ± 1.1	6.04 ± 0
SI	1.08	1.34	1.45	0.71	0.48	
**L-asparaginase (U/mL)**						
Cytotoxicity IC_50_ (U/mL)	2.16 ± 0.2	2.54 ± 0.3	4.95 ± 0.4	5.90 ± 0.4	7.59 ± 0.5	8.5 ± 0.5
SI	3.94	3.35	1.72	1.44	1.12	

HepG-2 (hepatocellular carcinoma), HeLa (cervical epitheloid carcinoma), Hep2 (epidermoid larynx carcinoma), MCF-7 (breast carcinoma), Caco2 (Colorectal adenocarcinoma), WI-38 (normal human lung fibroblast)

Figure Legands.

Standard doxorubicin inhibited the viability of cells of HeLa, MCF-7, HepG-2, Hep2, Caco2 and normal WI-38 cell lines by 91.6, 93.8, 93.7, 91.6, 84.9 and 92.1%; respectively. Doxorubicin showing very strong cytotoxic activity against both cancerous and non-cancerous cell lines with IC_50_ values of 5.57 ± 0.3, 4.50 ± 0.2, 4.17 ± 0.2, 8.54 ± 0.6, 12.49 ± 1.1 and 6.04 ± 0.4 µM against HeLa, HepG-2, MCF-7, Hep2, Caco2 and normal WI-38 cell lines; respectively (Table 9). Furthermore, the selectivity index, which indicates the cytotoxic selectivity of L-asparaginase (L-asparaginase safety) on HeLa, HepG-2, MCF-7, Hep2, and Caco2 cell lines was 3.94, 3.35, 1.72, 1.44 and 1.12; respectively. The value of selectivity index above or equal to 2 is of interest^66^. The results demonstrated that *Streptomyces rochei* subsp. *chromatogenes* NEAE-K purified L-asparaginase is a promising anticancer drug.

Sudarkodi and Sundar^67^ reported that L-asparaginase showed a good anticancer activity against HeLa and HepG-2 cell lines. The purified L-asparaginase showed a dose-depended cytotoxic effect on HeLa cells^68^. On the other hand, D’Souza *et al*.^69^ reported that L-asparaginase showed a significant cytotoxic activity against MCF-7, HeLa, HepG-2 and 3T3L1 cell lines. However, Moharib^70^ reported that L-asparaginase has higher cytotoxic activity against HepG-2 and HCT-116 than HeLa and MCF7 carcinoma cell lines. The anticancer activity of the purified L-asparaginase showed significant toxic activity toward HepG-2 cells, MCF-7 and HCT-116 cells^71^. Variations in the cytotoxic effects of L-asparaginase on different cell lines may be depend on the genetic nature of the microbial strain used for L-asparaginase production. The L-asparaginases obtained from various plant and microbial sources have different physiochemical and kinetic characteristics^72^.

## Materials and Methods

### Microorganism used in this study and cultural conditions

*Streptomyces* sp. strain NEAE-K used in this study was isolated from a soil sample obtained from Taif in Saudi Arabia. Isolation of *Streptomyces* sp. strain NEAE-K has taken place on the agar plates of starch nitrate medium. The plates have been incubated at 30°C for seven days. The isolate was preserved in 20% (v/v) glycerol as a spore suspension for subsequent studies.

### Evaluation of L-asparaginase production by agar plates assay method

The assessment of L-asparaginase production was performed using the agar plates of asparagine dextrose salts consisting of (%): Dextrose 0.2 g; K_2_HPO_4_ 0.1 g; L-asparagine 1.0 g; MgSO_4_ 0.05 g; Agar 2 g per 100 mL water; pH was adjusted to 6.8-7. Phenol red as an indicator of pH (0.009 per cent final concentration in ethanol) was added to the medium^73^ and sterilized. The plates were inoculated, inverted and incubated at 30 °C for 5–7 days. The production of L-asparaginase resulted in a rise in pH of culture medium causing changes in color of the pH indicator from yellow to pink. Isolate surrounded by pink zone was considered as L-asparaginase producing strain and were chosen for further studies. Plates of control were prepared as a dye-free medium.

### Inoculum preparation

The spore inoculum was made from a seven-day old culture which grew on a slope of starch-nitrate agar and the spores were suspended by a sterile loop in 10 mL sterile medium of production supplemented with 0.01% (v/v) of Tween 80^74^.

### Comparative assessment of substrates for L-asparaginase production and solid state fermentation

Local wheat bran and soy bean grains have been obtained from Mansoura local market, Egypt and were used as substrate for production of L-asparaginase. The grains were ground to produce around 1-3 mm of particle size. The efficacy of agro-industrial residues as a carbon source (wheat bran and soybean meal; separately and in combination) for the production of L-asparaginase was assessed. Solid state fermentation was performed in 250 mL Erlenmeyer flasks sets containing 10 g of each and mixed wheat bran and soybean meal were added in 1:1 proportions, to which freshly prepared asparagine dextrose salts broth medium was supplemented. The contents were thoroughly mixed and sterilized. The sterilized medium components were inoculated with 2 mL of inoculum to get the desired moisture content. The flasks contents were thoroughly mixed with a sterile wooden spoon and incubated in a stationary position at 30 °C for 5–7 days.

### Crude enzyme preparation

After incubation, the crude enzyme was extracted from the fermented substratum using a 90 mL sodium phosphate buffer (pH 7, 0.1 M). The fermented substratum and sodium phosphate buffer were homogenized with a constant stirring by agitation in a rotary shaker at 150 rpm for 1 h. The crude enzyme was centrifuged in a cooling centrifuge (at 4 °C) for 15 min at 5000 × *g*. The resulting supernatant was used for L-asparaginase activity assay.

### Assay of L-asparaginase activity

L-asparaginase activity was calculated from estimates of the quantities of ammonia released from L-asparagine hydrolysis by direct nesslerization using Wriston and Yellin^75^ method. One blank was prepared for each sample tested to exclude the quantity of ammonia generated throughout the microbial fermentations. One unit (U) of L-asparaginase is described as the quantity of the enzyme releasing 1 μmole of ammonia as a result of L-asparagine hydrolysis at 37 °C per minute using pH 8.6. The enzyme activity was expressed in terms of units per gram dry fermented substrate (U/gds).

### Assay of L-glutaminase

Imada *et al*.^76^ technique was used to estimate the L-glutaminase activity of both culture filtrate and purified *Streptomyces rochei* L-asparaginase using L-glutamine as substrate. The reaction mixture containing 0.5 mL of the enzyme preparation and 1.5 mL of 0.04 M L-glutamine (prepared in 0.05 M Tris-HCl buffer, pH 8.6). The reaction mixture tubes were incubated in a water bath shaker for 30 minutes at 37 °C. The reaction was stopped by adding 0.5 mL of 1.5 M Trichloroacetic acid (TCA). The blank was prepared by adding the enzyme after the addition of TCA, one blank has been conducted for each sample tested. The precipitated proteins were separated by centrifugation at 10,000 × *g* for 10 minutes. The amount of released ammonia was determined colorimetrically by direct nesslerization by adding 1 mL of Nessler’s reagent to tubes containing 0.5 mL of clear supernatant diluted with 7 mL of distilled water and incubated at room temperature for 20 min. The formation of the yellow color reflects the presence of ammonia and was measured against a blank at 450 nm using Optizen Pop–UV/Vis spectrophotometer. The amount of ammonia released was determined by using the standard curve of ammonium chloride.

### Spore chain morphology and cultural characteristics

The spore chain morphology of *Streptomyces* sp. strain NEAE-K was examined on ISP medium 4 cultured at 30 °C for 7–14 days. The dehydrated gold covered samples had been investigated for various magnifications with Jeol JSM-6360 LA Scanning Electron Microscope. The color of the aerial spores, pigmentation of the substrates mycelia and the production of diffusible pigments have been detected at ISP media 2–7 as illustrated by Shirling and Gottlieb^77^.

### Whole cell sugar analysis

Sugars were identified by High-Performance Liquid Chromatography (HPLC). The whole dried cells (50 mg) were hydrolyzed for 10 h at 100 °C in 1 mL of 2 mol/L trifluoroacetic acid in a sealed Pyrex tube. The residual acid was removed in a water-bath at 60 °C under a stream of N_2_ after the addition of methanol and taking the contents to dryness three successive times. The residue was dissolved by adding 1 mL of distilled water. Monosaccharides analysis in the extract of the whole-cell hydrolyzate was done with High-Performance Liquid Chromatography (HPLC, Agilent 1100 with Refractive Index Detector) using 4.6 × 250 mm Hypersil ASP-2 column with a mobile phase of 80: 20 acetonitrile-water and a flow rate of 0.4 mL min^−1^. The optical unit temperature and column were set at 40°C and 35°C; respectively. The injection volume of the standard and sample hydrolyzates was 10 μL.

The monosaccharides in the sample hydrolyzate was identified by comparing the obtained retention times with that of the standards for nine monosaccharides (fructose, xylose, mannose, arabinose, glucose, galactose and rhamnose) which injected in a concentration of 10 mg mL^−1^ under the same HPLC conditions.

### Physiological characteristics

The physiological features of *Streptomyces* sp. strain NEAE-K have been carried out according to the guidelines of Shirling and Gottlieb^77^. The strain’s ability to inhibit the growth of two yeasts “(*Candida albicans, Sacchromyces cerevisiae*), five strains of fungi (*Fusarium oxysporum, Aspergillus niger*, *Bipolaris oryzae*, *Alternaria solani, Rhizoctonia solani*) and five strains of bacteria (*Klebsiella*, *Pseudomonas aeruginosa, Staphylococcus aureus*, *Bacillus subtilis, Escherichia coli*)” was determined. The chitosanase^78^, asparaginase^73^ and uricase^79^ activities were also assessed.

### 16S rRNA analysis

The PCR reaction was conducted and the reaction mixture was purified and sequenced according to El-Naggar *et al*.^80^ methods. The neighbor-joining phylogenetic tree was generated using version 2.1 of MEGA4 software^30^.

### Selection of the significant variables using Plackett–Burman design

In the optimization of bioprocesses, Plackett–Burman design^81^ is commonly used to screen and assess the significant variables influencing the response. It reduces the experiments to a minimum by screening *n* variables in *n* + 1 runs. It is often used to study more than five independent variables. The design of Plackett-Burman can rapidly identify the key physicochemical variables that have large effects on the response of multiple independent variables in a very small number of experiments with regard to their main effects^82^. Plackett-Burman design disadvantage is that it does not describe the mutual interactions among the independent variables.

In the present study, Plackett–Burman experimental design comprised of 20 experiments was used to screen sixteen independent factors for their impacts on the production of L-asparaginase including: A (incubation time; days), B (moisture content; %), C (inoculum size; %), D (pH), E (temperature; °C), F (wheat bran+soybean meal; 1:1 g), G (dextrose; g/L), H (fructose; g/L), J (L-asparagine; g/L), K (K_2_HPO_4_; g/L), L (KNO_3_; g/L), M (yeast extract; g/L), N (MgSO_4_.7H_2_O; g/L), O (NaCl; g/L), P (FeSO_4_. 7H_2_O; g/L) and Q (CaCl_2_; g/L) (Table 2). Each variable is represented at two levels, high (+) and low (−). Plackett-Burman experimental design is based on the first order model:

3 $$Y={\beta }_{0}+\Sigma {\beta }_{i}{X}_{i}$$

where, Y is production of L-asparaginase, β_0_ is the intercept of the model and *β*_*i*_ is the linear coefficient, and X_i_ is the independent variable level.

### Central composite design (CCD)

CCD has been used to determine the optimized levels and to identify possible interactions between different significant variables. In the present study, the design comprised of 20 experiments where a three independent variable was tested at five different levels (−1.68, −1, 0, 1, 1.68). All experiments were conducted three times and the average of L-asparaginase activity attained has been taken as a response (Y). The obtained results of CCD were analyzed via the second order polynomial equation:

4 $$Y={\beta }_{0}+\sum _{i}{\beta }_{i}{X}_{i}+\sum _{ii}{\beta }_{ii}{{X}_{i}}^{2}+\sum _{ij}{\beta }_{ij}{X}_{i}{X}_{j}$$

In which Y is the predicted response, X_i_ is the independent variable coded level, β_ij_ is the interaction coefficients, β_0_ is the regression coefficients, β_ii_ is the quadratic coefficients and β_i_ is the linear coefficient.

### Statistical analysis

The experimental designs and statistical analysis have been carried out using the software version 7 of design expert for Windows. The 3D surface plots have been constructed with version 8 of STATISTICA software.

### Purification of L-asparaginase from *Streptomyces rochei* subsp. *chromatogenes* NEAE-K

L-asparaginase purification has been carried out using the method of El-Naggar *et al*.^64^. L-asparaginase activity was calculated by estimates of the quantities of ammonia released from L-asparagine hydrolysis by direct nesslerization using Wriston and Yellin^75^ method. Protein was estimated according to the method of Lowry *et al*.^83^.

### Enzyme monomeric molecular weight determination

In accordance with the Laemmli^62^ method, the monomeric molecular weight of the purified L-asparaginase was defined by SDS-PAGE with protein marker ranges between 9 and 178 kDa.

### The cell lines and cell cultures

The five human tumor cell lines used in this study were HepG-2 (hepatocellular carcinoma), HeLa (cervical epitheloid carcinoma), Hep2 (epidermoid larynx carcinoma), MCF-7 (breast carcinoma) and Caco2 (Colorectal adenocarcinoma) and the normal cell line (WI-38 cell line) were obtained from ATCC via VACSERA company, Cairo, Egypt. The RPMI-640 medium supported by 10% fetal bovine serum was used to cultivate the cell lines. 100 μg/mL of streptomycin and 100 U/mL of penicillin were added as antibiotics. Cells were maintained at 37 °C in a 5% CO_2_ incubator.

### MTT colorimetric assay

The previously mentioned cell lines have been used to assess L-asparaginase’s inhibitory effects on tumor cell lines using the yellow tetrazolium bromide (MTT) test. At density of 1×10^4^ cells/well, the cell lines were seeds in complete medium in a 96-well plate and incubated at 37°C in a 5% CO_2_ incubator. After incubation for 48 hours, the tumor cell lines have been treated with various concentrations of L-asparaginase (10, 7.5, 5, 3.75, 2.5, 1.875, 1.25 U/mL) and doxorubicin (DOX) (100, 50, 25, 12.5, 6.25, 3.125, 1.56 µM) suspended in serum free medium and incubated for 24 at 37°C. The drugs were added to the same medium used for seeding. After L-asparaginase and doxorubicin treatment, 20 μL of the MTT solution at a concentration of 5 mg/mL were supplied to each well and incubated at 37°C for an additional 4 h. To dissolve the formed purple formazan, dimethyl sulfoxide (DMSO, sigma Co., St. Louis, USA) was added to each well in a volume of 100 µL. The colorimetric assay was assessed with a plate reader (EXL 800, USA) and recorded at 570 nm (OD). The effects of the L-asparaginase and doxorubicin on the different cell lines were expressed as the % of cytotoxicity (cell growth inhibition) using the following formula^84^:

$$ \% \,{\rm{cytotoxicity}}=100- \% \,{\rm{cell}}\,{\rm{viability}}$$

where % cell viability = (A_570 nm_ of treated cells)/(A_570 nm_ of controlled) × 100

### Selectivity index (SI)

The anticancer selectivity was expressed as the following: SI = IC_50_ of the tested enzyme in a normal cell line/IC_50_ of the tested enzyme in tumor cell line, where IC_50_ is the tested enzyme concentration required to kill 50% of the cells^85^. The IC_50_ value was calculated with Graph Pad Prism software (version 5).

## Conclusion

*Streptomyces* sp. strain NEAE-K has been isolated from a soil sample obtained from Taif in Saudi Arabia and identified as *Streptomyces rochei* subsp. *chromatogenes* NEAE-K. Statistical designs were used to optimize L-asparaginase production under solid state fermentation conditions. The maximum L-asparaginase production was found to be 175.96 U/gds by using the following medium compositions (g/L): L-asparagine 7, yeast extract 3, wheat bran + soybean meal 15, fructose 4, dextrose 2.6, CaCl_2_ 0.01, FeSO_4_. 7H_2_O 0.02, KNO_3_ 1, K_2_HPO_4_ 2, MgSO_4_.7H_2_O 0.5, NaCl 0.1. The optimum moisture content was 50%, pH 6.7, inoculum 2 mL and incubation temperature 37°C for 7 days. L-asparaginase purification was performed with a final purification fold of 16.18. The monomeric molecular weight of the purified L-asparaginase was found to be 64 kDa. The *in vitro* effect of L-asparaginase results demonstrated that the L-asparaginase strongest cytotoxic effect was exerted on the HeLa cell line (IC_50_ = 2.16 ± 0.2 U/mL). The IC_50_ values ranged from 2.16 ± 0.2 to 7.59 ± 0.5 U/mL after 24 h.

## Supplementary information

Supplementary Information.
