# Supplementary material for: Bioprocess development for L-asparaginase production by Streptomyces rochei, purification and in-vitro efficacy against various human carcinoma cell lines
Source: Sci Rep. 2020 May 14;10:7942. doi: 10.1038/s41598-020-64052-x (PMC7224186; doi:10.1038/s41598-020-64052-x)

**Bioprocess development for L-asparaginase production by *Streptomyces rochei*, purification and *in-vitro* efficacy against various human carcinoma cell lines**

**Noura El-Ahmady El-Naggar*, Nancy M. El-Shweihy**

Department of Bioprocess Development, Genetic Engineering and Biotechnology Research Institute, City of Scientific Research and Technological Applications, Alexandria, Egypt

**Corresponding Author’s information**

**Dr. Noura El-Ahmady Ali El-Naggar**

**Address:**

Bioprocess Development Department,

Genetic Engineering and Biotechnology Research Institute,

City of Scientific Research and Technological Applications,

New Borg El- Arab City, 21934, Alexandria, Egypt

**Tel:** (002)01003738444

**Fax:** (002)03 4593423

**E-mail:** nouraelahmady@yahoo.com

**Complete gel for Figure 10**


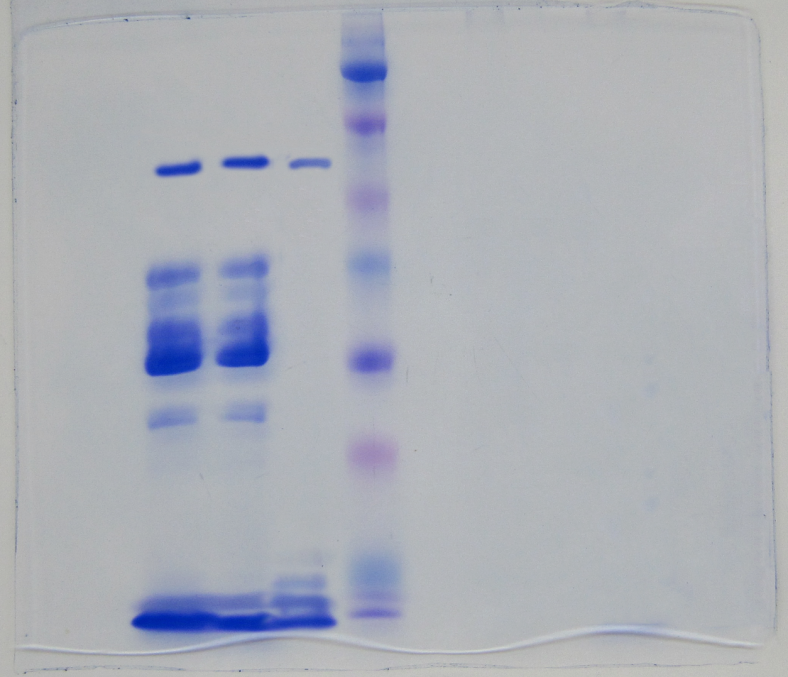

Supplement: Supplementary file 1 — Supplementary Information. [file 41598_2020_64052_MOESM1_ESM.docx]
